# A Comparison of Chronic Periodontitis in HIV-Seropositive Subjects and the General Population in the Ga-Rankuwa Area, South Africa

**DOI:** 10.1155/2012/620962

**Published:** 2012-08-29

**Authors:** Razia Khammissa, Liviu Feller, Mario Altini, Paul Fatti, Johan Lemmer

**Affiliations:** ^1^Department of Periodontology and Oral Medicine, University of Limpopo, Medunsa Campus, Pretoria 0204, South Africa; ^2^Division of Oral Pathology, University of the Witwatersrand, Johannesburg 2050, South Africa; ^3^School of Statistics and Advanced Science, University of the Witwatersrand, Johannesburg 2050, South Africa

## Abstract

The effect of HIV infection on the prevalence and the rate of progression of chronic periodontitis is not clear. The aim of this study was to compare parameters associated with the severity of chronic periodontitis in terms of periodontal probing depths, gingival recession, plaque indexes, and bleeding indexes of HIV-seropositive subjects and healthy age-matched control subjects, and of HIV-seropositive subjects on highly active antiretroviral therapy and those not receiving such treatment. Two cohorts of subjects with chronic periodontitis were recruited for this study over a period of six months. There were 30 HIV-seropositive subjects, and 30 control subjects. Periodontal probing depths, gingival marginal recession, plaque indexes, and bleeding indexes were compared by HIV serostatus, the use of highly active antiretroviral therapy, and CD4+ T-cell counts. All participants were black persons between the age of 18 and 45 and were of a similar socioeconomic status and age. The results of this study indicate that chronic periodontitis in HIV-seropositive subjects is similar in terms of mean periodontal probing depth, gingival marginal recession, plaque index, and bleeding index to that in healthy age-matched control subjects, and a low CD4+ T-cell count does not appear to be a risk factor for increased severity of chronic periodontitis.

## 1. Introduction

The relationship between chronic periodontitis and HIV infection is not clear and considerable differences of opinion exist regarding the prevalence of chronic periodontitis among HIV-seropositive subjects [1, 2]. Microbiological studies have failed to detect any major differences in the subgingival microbial flora of HIV-seropositive subjects with chronic periodontitis compared to HIV-seronegative controls [3, 4], and the humoral immune response to the periodontopathic bacteria is similar in both groups [5]. 

Some authors reported a higher prevalence of periodontal attachment loss and a more rapid progression of periodontal disease over time in HIV-seropositive subjects compared to HIV-seronegative controls [6–8]. A great proportion of the loss of periodontal attachment seen in HIV-seropositive subjects with chronic periodontitis is said to be owing to localized gingival marginal recession rather than to the formation of deep periodontal pockets as in HIV-seronegative subjects [7, 9–11]. However, other studies failed to document differences between the natural course of chronic periodontitis in HIV-seropositive subjects compared with the course in HIV-seronegative subjects with chronic periodontitis [12, 13]. 

The considerable differences of opinion about the natural course of chronic periodontitis in HIV-seropositive subjects may cause corresponding confusion with regard to their periodontal treatment. The aim of this study was to compare parameters associated with the severity of chronic periodontitis in terms of periodontal probing depths, gingival recession, plaque indexes, and bleeding indexes of HIV-seropositive subjects and control subjects and of HIV-seropositive subjects on highly active antiretroviral therapy and those not receiving such treatment.

## 2. Materials and Methods

### 2.1. Subject Population

Approval of this study was obtained from the Ethics Committees of the Universities of Limpopo and of the Witwatersrand, Johannesburg. Two cohorts of subjects with chronic periodontitis were recruited for this study over a period of six months: thirty HIV-seropositive subjects and 30 control subjects presumed to be HIV-seronegative and apparently in good health. All these patients did not receive periodontal treatment before recruitment. 

The term “apparently healthy subject” in this paper refers to someone who according to his medical history is in a state of good physical and mental well being, is not pregnant, not diabetic, and is not known to have HIV infection or any other condition of immune dysregulation or any other physical condition that is known to be associated with increased risk of periodontal disease. In addition, these apparently healthy subjects should not at the time of periodontal examination be taking any medication that may adversely affect the periodontium such as calcium channel blockers or phenytoin. After explanation of the purpose of the study, all gave their informed consent to participate. There were 34 females and 26 males, all black persons between the ages of 18 and 45 years. 

Of the 30 HIV-seropositive subjects, 16 were receiving highly active antiretroviral treatment (HAART) and 14 were not receiving such treatment (HAART-naïve). The serostatus of all HIV-seropositive subjects had been confirmed by enzyme-linked immunosorbent assay (ELlSA) and western blot. CD4+ T-cell counts were performed for 13 of the 30 HIV-seropositive subjects who had given informed consent. 

### 2.2. Periodontal Health Status

Chronic periodontitis was diagnosed by clinical and radiographic examination by a single clinician. Subjects were diagnosed with chronic periodontitis when at least three tooth sites had periodontal probing depth ≥5 mm and/or had measurable gingival marginal recession, and where there was radiographic evidence of loss of alveolar bone height. 

Periodontal probing depths (PPD), gingival marginal recession (GR), plaque indexes (PI), and bleeding indexes (BI) were measured. PPD refers to the distance from the gingival margin to the location of the tip of a periodontal probe inserted in the pocket with moderate force [14] and gingival marginal recession refers to the distance from the cementoenamel junction to the location of the apically displaced gingival margin [15]. PPD was measured at six sites per tooth (mesiobuccally, midbuccally, distobuccally, distolingually, midlingually and mesiolingually) and GR was measured at two sites per tooth, buccal and lingual, excepting third molars and residual roots. The PPD and GR measurements were done with a periodontal probe with Williams markings. 

PI and BI scored the presence or absence of plaque and bleeding on probing, respectively: presence scored 1 and absence scored 0 [14, 16]. BI was calculated as the sum of bleeding sites (any bleeding on probing around the circumference of a tooth was counted as a site) divided by the number of teeth present in the mouth, excluding third molars and residual roots, and converting the quotient into a percentage. Disclosing solution was used to display bacterial plaque and PI was calculated by dividing the sum of plaque present at four tooth sites (mesial, buccal, distal, and lingual) by the number of teeth, multiplied by four, and converting the quotient into a percentage [17, 18].

No attempt was made to differentiate sites of active periodontitis showing suppuration or bleeding on probing from inactive sites with evidence of increased PPD. 

For the purpose of this study, a mean PPD value per mouth and a mean GR value per mouth were calculated to provide a straight forward comparison of periodontal status of the studied and the control group of patients. 

### 2.3. Statistical Analysis

All data were entered into the Microsoft Excel program and analyzed using its data analysis package. ANOVA, *t-*tests, Pearson's correlation coefficients, and histograms were computed to conduct statistical hypothesis tests and to explore associations. *P* values of <0.05 were regarded as statistically significant. 

Single factor analysis of variance was used to test for differences between the periodontal indices of the control subjects and the two HIV-seropositive subject groups. The two-sample *t-*test was used to test for differences between the periodontal indexes of the HIV-seropositive HAART-naïve subjects and of the HIV-seropositive subjects using HAART. The Pearson correlation coefficient was used to test for significant relationships between the periodontal indexes of the HIV-seropositive subjects and the log CD4+ T-cell count. 

A log transformation of the CD4+ T-cell count was performed to correct for its skew distribution. 

## 3. Results

The mean number of teeth per mouth of the group of HIV-seropositive subjects (29 teeth) and of the group of the control subjects (28 teeth) was similar. Periodontal probing depth (PPD), gingival recession (GR), plaque index (PI), and bleeding index (BI) were compared by HIV-serostatus, the use of HAART, and CD4+ T-cell counts. CD4+ T-cell counts were stratified into the following groups: CD4+ T-cell count <200 cells/mm^3^, CD4+ T-cell counts 200–500 cells/mm^3^, and CD4+ T-cell count >500 cells/mm^3^. HAART was defined as the use of at least two nucleoside reverse transcriptase inhibitors with either a nonnucleoside reverse transcriptase inhibitor or a protease inhibitor. All participants were black persons from the Ga-Rankuwa area in South Africa and were of a similar socioeconomic status and age. CD4+ T-cell counts were available for eight HIV-seropositive subjects using HAART and for five HAART-naïve subjects. There were only eight subjects that admitted to smoking. 

When all participants were evaluated, no association was found between HIV-serostatus and periodontal indexes. HIV-seropositive and control subjects with chronic periodontitis had similar mean PPD, GR, PI, and BI (Table 1), and gender had no influence on the results (data not shown). The mean periodontal indexes for PPD, GR, PI, and BI were compared between the two groups of HIV-seropositive and the control subjects using ANOVA. None of them were significant at the *P* < 0.05 level. 

When periodontal indexes of HIV-seropositive subjects using HAART were compared to the periodontal indexes of HAART-naïve subjects, using the *t*-test, there was no statistical differences regarding mean GR, PI, and BI.However, the mean PPD in HAART-naïve seropositive subjects was slightly greater than in HIV-seropositive subjects using HAART (*r* = 0.01), 3.36 mm and 3.07 mm, respectively (Table 2). 

The Pearson correlation coefficient of mean PPD in relation to log CD4+ T-cell count in the HIV-seropositive HAART-naïve group of subjects showed a significant negative correlation (*r* = −0.947), but there was no significant correlation between the mean GR values and the log CD4+ T-cell counts in the same group. For the HIV-seropositive subjects using HAART the Pearson correlation coefficient test failed to show significant statistical relationships between log CD4+ T-cell count and mean PPD and between log CD4+ T-cell count and mean GR*.*

Unexpectedly, the mean CD4+ T-cell count was higher in the HAART-naïve group of subjects than in HIV-seropositive subjects using HAART (*r* = 0.32) (Table 3). When the average log CD4+ T-cell counts of HAART-naïve HIV-seropositive subjects were compared, using the *t-*test, to the CD4+ T-cell counts of HIV-seropositive subjects using HAART, the difference between them was not significant at the 5% level. This finding can be explained by the fact that the CD4+ T-cell counts were available only for a small number of subjects, and by the fact that HIV-seropositive subjects treated in provincial hospitals in South Africa generally receive HAART only after their CD4+ T-cell count has fallen below 200 cells/mm^3^. 

Evaluation of the impact of smoking on PPD, GR, PI, and BI was not an aim of this study. However, in view of the well-established association between smoking and severity of chronic periodontitis, we compared these indexes by HIV-serostatus in the non-smoking subjects of the population study (27 nonsmoking HIV-seropositive subjects and 25 non-smoking control subjects), but not in the smoking subjects as the samples were too small (3 smoking HIV-seropositive subjects and 5smoking control subjects). There was no significant difference between the non-smoking HIV-seropositive and control subjects in any of these indexes.

## 4. Discussion

This study demonstrates that both HIV-seropositive and apparently healthy subjects with chronic periodontitis have similar mean PPD, GR, PI, and BI measurements and that HIV infection does not carry with it a greater risk for accelerated periodontal attachment loss [2, 19]. This conforms with other studies that documented similar clinical manifestations and natural courses of chronic periodontitis in HIV-seropositive and -seronegative subjects [12, 13, 20, 21].

The results of this study indicate that the reported general increase in the prevalence of fungal and viral microorganisms observed in periodontal pockets of HIV-seropositive subjects with chronic periodontitis compared to HIV-seronegative subjects with chronic periodontitis [22–25] has no influence on the periodontal health status, in spite of the potential of these microorganisms to exacerbate the inflammatory processes and suppress the immune responses in the periodontal tissues [26]. It is also evident that the reduction in the number of Langerhans cells in the pocket epithelium in HIV-seropositive subjects [27, 28] and the consequent local immune impairment does not play any particular role in the development and progression of chronic periodontitis. 

Therefore, there would appear to be no indication for instituting different treatment modalities for chronic periodontitis in HIV-seropositive subjects and in immunocompetent subjects. If indeed some HIV-seropositive subjects demonstrate increased levels of periodontal tissue destruction, this may be the result of periodontal disease activity that took place before the onset of HIV infection. This could be determined by the study of reliable pre-HIV infection periodontal records [29]. 

The mean GR was similar in HIV-seropositive and healthy subjects, in HIV-seropositive subjects using HAART, and in HIV-seropositive HAART-naïve subjects, and also regardless of lower or higher CD4+ T-cell counts. This does not agree with the findings of McKaig et al. (2000) [10] and Lamster et al. (1997) [30] who found a negative correlation between CD4+ T-cell count and increased frequency of recession. McKaig et al., (2000) [10] reported that recession in HIV-seropositive subjects with chronic periodontitis is more likely to occur in association with low CD4+ T-cell counts (<200 cells/mm^3^) than with higher CD4+ T-cell counts (200–499 cells/mm^3^). 

As evident from the results of this study, HIV-seropositive subjects with chronic periodontitis do not have an increase either in the number of sites of gingival marginal recession or in the severity of gingival marginal recession; and a low CD4+ T-cell count is not a risk factor for increased frequency or severity of GR.

Recent studies have shown that the severity of chronic periodontitis is decreased in HIV-seropositive subjects and that there is a positive correlation between clinical attachment levels and the CD4+ T-cell counts; lower CD4+ T-cell counts are associated with reduced periodontal probing depth and with lesser degrees of recession measurements [31, 32]. 

The profound suppression of immunity in HIV disease does not seem to increase the risk of development of chronic periodontitis. The diminution of the CD4+ T-cell count, dysregulation of the cytokine network, and qualitative defects of macrophages, monocytes, polymorphonuclear leukocytes, dendritic cells, and T lymphocytes do not predispose HIV-seropositive subjects to periodontopathic infection [33, 34]. The profound HIV-associated immune suppression also does not appear to predispose to delayed wound healing of the oral tissues [35], to increased incidence of wound infection of periodontal or other oral surgery [36], or to plaque-induced periodontal tissue destruction [12, 13, 20].

Since the periodontal tissue destruction in chronic periodontitis is mediated mainly by host-derived cellular immune responses [37], and since these mechanisms are to a great extent suppressed in HIV infection, HIV-seropositive subjects with chronic periodontitis may be expected to show reduced rather than exaggerated periodontal tissue destruction, compared to immunocompetent subjects with chronic periodontitis. Moreover, active periodontal disease associated with periodontal attachment loss is related mainly to a Th1 cytokine profile [38, 39]. However, in advanced HIV disease, in the absence of HAART, there is a dysregulation in the cytokine network characterized by a shift from Thl predominant cytokines to a Th2 cytokine profile [40] that is less associated with periodontal tissue destruction. This reinforces the concept that HIV-related immune dysregulation may not contribute to the development of chronic periodontitis but in fact is associated with reduced periodontal tissue destruction. 

Taking into consideration the immune suppression associated with HIV infection one would have expected that HIV-seropositive subjects with low CD4+ T-cell counts would show higher frequencies of infection with periodontopathic bacteria and an increased incidence of wound infection following oral surgery, compared to HIV-seropositive subjects. However, this is not the case. HIV-seropositive and -seronegative subjects show similar types and quanta of periodontopathic bacteria in their subgingival microbiota, similar incidences of infection of surgical wound, and similar wound healing capacity [4, 29]. 

HIV-seropositive subjects, however, demonstrate bacterial species in their subgingival microbiota that are not usually associated with periodontal disease. These opportunistic microorganisms including *E. faecalis, A. baumannii, *and *Pseudomonas aeruginosa *are probably associated with HIV-related immunosuppression. The fact that HIV-seropositive subjects are frequently treated in a hospital environment might account for the presence of unusual opportunistic microorganisms [31]. 

The process of wound healing is complex, involving interaction between macrophages, dendritic cells, polymorphonuclear leukocytes, epithelial cells, fibroblasts, osteoblasts, cytokines, and growth factors [41]. While macrophages and growth factors are the driving force behind the process of wound healing [41], CD4+ T-cells do not play a critical role [42]. Consequently, in spite of some impairment in the function of macrophages and alteration in the cytokine network, HIV-seropositive subjects do not demonstrate impaired wound healing capacity [43] or increased incidence of wound infection following oral surgery [35]. 

There is an inverse relation between the CD4+ T-cell count and the frequency of HIV-associated oral lesions, in particular when the CD4+ T-cell count is lower than 200 cells/mm^3^ regardless of the use of HAART [44]. However, HIV-seropositive subjects using HAART have a significantly lower prevalence of HIV-associated oral lesions compared to HAART-naïve HIV-seropositive subjects [44]. 

Chronic periodontitis is similar to other oral lesions in this respect. The use of HAART in HIV-seropositive subjects has brought about a decrease in the prevalence and severity of chronic periodontitis [11, 44]. HIV-seropositive subjects with chronic periodontitis using HAART demonstrate reduced counts of periodontopathic bacteria in their subgingival plaque, and their periodontal tissues show reduced inflammation and periodontal attachment loss, compared to HIV-seronegative subjects with chronic periodontitis [34]. 

Even severely immunocompromised HIV-seropositive subjects with chronic periodontitis who are using HAART and with low CD4+ T-cell counts demonstrate similar periodontopathic bacteria in their subgingival microbiota to HIV-seronegative subjects with chronic periodontitis of a comparable degree [33]. It is possible that HAART-associated reconstitution of components of the immune response is sufficient to control colonization by the periodontopathic pathogens, even though the CD4+ T-cell count remains low [33, 34]. It is also possible that one or more of the drugs in the cocktail constituting HAART have anti-inflammatory and/or antibacterial properties that act synergistically with the hosts' partially reconstituted immune mechanisms in controlling the periodontopathic bacteria [34].

This study has a few limitations. Firstly, the control group comprised subjects of unconfirmed, presumably HIV-seronegative status, and this status could not be confirmed owing to the refusal of the subjects to consent to free HIV testing. Secondly, of the HIV-seropositive cohort of 30 subjects, the CD4+ T-cell count of only 13 subjects were known. The remaining 17 subjects would not consent to free CD4+ T-cell count testing.

In the semirural community of Ga-Rankuwa, South Africa, refusal of any form of testing in relation to HIV disease, even if offered free of charge, is common and is probably related to the perceived stigma associated with HIV disease and to the possibility that HIV-seropositive subjects may be shunned by their communities. Consequently, there is reluctance to learn one's own HIV-serostatus, and when the HIV-serostatus is known, to denial. 

In a study investigating necrotizing periodontal diseases (NPD) in HIV-seropositive and—seronegative subjects in the same cohort as in our study [45], 35% of subjects with NPD and unaware of their HIV-serostatus refused to test their HIV-serostatus in spite of strong recommendation and detailed explanation on the well-established association between NPD and HIV infection in the cohort. Even in research into oral disease not commonly associated with HIV infection, the offer of voluntary serostatus testing for HIV infection is invariably refused.

According to Statistics South Africa, an estimated 16.2% of South Africans between the ages of 15 and 49 years are HIV seropositive [46]. Therefore, although scientific proof of HIV-seronegativity would obviously be preferable, statistically an estimated 83.8% of control subjects in this study were likely to be HIV-seronegative. There were no statistically significant differences between the periodontal indexes of HIV-seropositive subjects all of whom had chronic periodontitis, and of control subjects all of whom had chronic periodontitis. 

CD4+ T-cell counts were available for only 13 HIV-seropositive subjects (eight subjects using HAART and five HAART-naïve subjects). With such a small cohort, the statistical analysis of CD4+ T-cell counts relative to periodontal indexes could have been skewed. In this study, the correlation coefficient of PPD with CD4+ T-cell count in the HIV-seropositive HAART-naïve group of subjects was significantly negative (*P* < 0.01). This differs from other studies [32] that documented a positive correlation between CD4+ T-cell counts and PPD measurements in HIV-seropositive subjects and that HIV-seropositive subjects with healthy periodontium that are using HAART have lower CD4+ T-cell counts, while HIV-seropositive subjects with chronic periodontitis using HAART, have higher CD4+ T-cell counts [31, 33]. These differences between the results of our study and the results of other studies may be attributed to our small sample of subjects with known CD4+ T-cell counts. 

In this study, the mean CD4+ T-cell counts found in HIV-seropositive HAART-naïve subjects (257 cells/mm^3^) was higher than in HIV-seropositive subjects using HAART (172 cells/mm^3^) (Table 3). This seemingly paradoxical finding could very well be attributed to skewed statistics associated with the small number of subjects with known CD4+ T-cell counts. However, on second consideration, this finding could be real. In South Africa, many people with oral conditions suggestive of HIV disease refuse to undergo serological testing for HIV and often prefer to be treated by traditional healers. As a result, HIV infection is often diagnosed and HAART is often introduced late in the course of the disease when the CD4+ T-cell count has already fallen very low (200 cells/mm^3^). In addition, in the South African context, HIV-seropositive subjects, who depend on provincial (governmental) services for their medical care, are subject to an official policy ruling that HAART may start only when their CD4+ T-cell count has fallen to 200 cells/mm^3^ or below. Under these circumstances, in practice, HAART is initiated only when the CD4+ T-cell count has fallen substantially below 200 cells/mm^3^. It is well established that starting HAART when the CD4+ T-cell count is very low will lead to a lower level of reconstitution of CD4+ T-cell numbers compared to the achieved level of reconstitution of CD4+ T-cell numbers when starting HAART at a higher CD4+ T-cell count. Hence, this seemingly paradoxical finding that the HIV-seropositive subjects using HAART in this study had a lower CD4+ T-cell count compared to the CD4+ T-cell count of HIV-seropositive HAART-naïve subjects.

In this study, the mean PPD in the HAART-naïve HIV-seropositive subjects with chronic periodontitis was slightly greater than in HIV-seropositive subjects using HAART (*r* = 0.01); the mean CD4+ T-cell count in the group of HIV-seropositive subjects using HAART was lower than in the group of HIV-seropositive HAART-naïve subjects. This conforms to other studies that report a positive correlation between PPD measurements and CD4+ T-cell counts [31, 32].

## 5. Conclusion

Chronic periodontitis in HIV-seropositive subjects is similar in terms of mean PPD, GR, PI, and BI to that in presumably healthy aged-matched control subjects, and a low CD4+ T-cell count does not appear to be a risk factor for increased frequency or severity of chronic periodontitis.

## Figures and Tables

**Table 1:** Epidemiological features and periodontal indexes of HIV-seropositive subjects with chronic periodontitis and control subjects with chronic periodontitis.

	Presumably HIV-seronegative control subjects with chronic periodontitis	HIV-seropositive subjects with chronic periodontitis
Females	14	20
Males	16	10
Smokers	5	3
Mean periodontal probing depths	3.196 mm	3.205 mm
STDEV*	0.58	0.32
Mean gingival recession	1.53 mm	1.66 mm
STDEV*	0.89	0.71
Number of gingival recession sites	195	202
STDEV*	5.11	5.46
Mean plaque index	75.2%	75.6%
STDEV*	23.6	20.16
Mean bleeding index	50.3%	47.3%
STDEV*	19.4	23.25

*STDEV: standard deviation.

**Table 2:** Epidemiological features, periodontal indexes, and immunological indexes of HIV-seropositive subjects using HAART of HAART-naïve HIV-seropositive subjects and of control subjects. All subjects had chronic periodontitis.

	Presumably HIV-seronegative control subjects with chronic periodontitis	HIV-seropositive subjects using HAART with chronic periodontitis	HAART-näive HIV-seropositive subjects with chronic periodontitis
Females	14	12	8
Males	16	4	6
Smokers	5	2	1
Mean periodontal probing depths	3.196	3.069	3.359
STDEV*	0.58	0.28	0.30
Mean gingival recession	1.53	1.67	1.64
STDEV*	0.89	0.79	0.64
Number of gingival recession sites	195	100	102
STDEV*	5.11	4.14	3.56
Mean plaque index	75.2%	71.9%	79.3%
STDEV*	23.6	20.4	26.1
Mean bleeding index	47.3%	42.5%	52.1%
STDEV*	23.25	18.3	22.1
Mean CD4+ count	Not available	171.63 cell/mm³	257.44 cell/mm³
STDEV*		108.94	171.25

*STDEV: standard deviation.

**Table 3:** The categories of CD4+ T-cell levels of the HIV-seropostive subjects using HAART and HIV-seropositive HAART-naïve subjects.

	HIV-seropositive subjects using HAART with chronic periodontitis	HIV-seropositive HAART-naïve subjects with chronic periodontitis
CD4+ count <200	4	2
CD4+ count 200–500	4	2
CD4+ count >500	0	1
Mean CD4+ count	172 cell/mm³	257 cell/mm³

## References

[1] Holmstrup P, Glick M (2002). Treatment of periodontal disease in the immunodeficient patient. *Periodontology 2000*.

[2] Stanford TW, Rees TD (2003). Acquired immune suppression and other risk factors/indicators for periodontal disease progression. *Periodontology 2000*.

[3] Zambon JJ, Reynolds HS, Genco RJ (1990). Studies of the subgingival microflora in patients with acquired immunodeficiency syndrome. *Journal of Periodontology*.

[4] Moore LVH, Moore WEC, Riley C, Brooks CN, Burmeister JA, Smibert RM (1993). Periodontal microflora of HIV positive subjects with gingivitis or adult periodontitis. *Journal of Periodontology*.

[5] Yeung SCH, Taylor BA, Sherson W (2002). IgG subclass specific antibody response to periodontopathic organisms in HIV-positive patients. *Journal of Periodontology*.

[6] Tomar SL, Swango PA, Kleinman DV, Burt BA (1995). Loss of periodontal attachment in HIV-seropositive military personnel. *Journal of Periodontology*.

[7] Robinson PG, Sheiham A, Challacombe SJ, Zakrzecwska JM (1996). The periodontal health of homosexual men with HIV infection: a controlled study. *Oral Diseases*.

[8] Robinson PG, Boulter A, Birnbaum W, Johnson NW (2000). A controlled study of relative periodontal attachment loss in people with HIV infection. *Journal of Clinical Periodontology*.

[9] Robinson PG (1997). Treatment of HIV-associated periodontal diseases. *Oral Diseases*.

[10] McKaig RG, Patton LL, Thomas JC, Strauss RP, Slade GD, Beck JD (2000). Factors associated with periodontitis in an HIV-infected Southeast USA study. *Oral Diseases*.

[11] Ryder MI (2002). An update on HIV and periodontal disease. *Journal of Periodontology*.

[12] Scheutz F, Matee MI, Andsager L (1997). Is there an association between periodontal condition and HIV infection?. *Journal of Clinical Periodontology*.

[13] Matee M, Nguvumali H, Lembariti B, Kalyanyama B, Shubi F, Scheute F (1999). HIV infection, dental treatment demands and needs among patients seeking dental services at the Muhimbili Medical Centre in Dar-es-Salaam, Tanzania. *International Dental Journal*.

[14] Papapanou PN, Lindhe J, Lindhe J, Lang NP, Karring T (2008). Epidemiology of periodontal diseases. *Clinical Periodontology and Implant Dentistry*.

[15] Wennström JL, Zucchelli G, Pini Prato GP, Lindhe J, Lang NP, Karring T (2008). Mucogingival therapy—periodontal plastic surgery. *Clinical Periodontology and Implant Dentistry*.

[16] Ainamo J, Bay I (1975). Problems and proposals for recording gingivitis and plaque. *International Dental Journal*.

[17] O’Leary TJ, Drake RB, Naylor JE (1972). The plaque control record. *Journal of Periodontology*.

[18] Slavi GE, Lindhe J, Lang NP, Lindhe J, Lang NP, Karring T (2008). Examination of patients with periodontal disease. *Clinical Periodontology and Implant Dentistry*.

[19] Khongkunthian P, Grote M, Isaratanan W, Piyaworawong S, Reichart PA (2001). Oral manifestations in HIV-positive adults from Northern Thailand. *Journal of Oral Pathology and Medicine*.

[20] Holmstrup P, Westergaard J (1994). Periodontal diseases in HIV-infected patients. *Journal of Clinical Periodontology*.

[21] Persson RE, Hollender LG, Persson GR (1998). Alveolar bone levels in AIDS and HIV seropositive patients and in control subjects. *Journal of Periodontology*.

[22] Brady LJ, Walker C, Oxford GE, Stewart C, Magnusson I, McArthur W (1996). Oral diseases, mycology and periodontal microbiology of HIV-1-infected women. *Oral Microbiology and Immunology*.

[23] Chattin BR, Ishihara K, Okuda K, Hirai Y, Ishikawa T (1999). Specific microbial colonizations in the periodontal sites of HIV- infected subjects. *Microbiology and Immunology*.

[24] Mardirossian A, Contreras A, Navazesh M, Nowzari H, Slots J (2000). Herpesviruses 6, 7 and 8 in HIV- and non-HIV-associated periodontitis. *Journal of Periodontal Research*.

[25] Contreras A, Mardirossian A, Slots J (2001). Herpesviruses in HIV-periodontitis. *Journal of Clinical Periodontology*.

[26] Feller L, Meyerov R, Lemmer J (2007). The association between human herpes viruses and periodontal disease, part 2. *South African Dental Journal*.

[27] Chou LL, Epstein J, Cassol SA, West DM, He W, Firth JD (2000). Oral mucosal Langerhans’ cells as target, effector and vector in HIV infection. *Journal of Oral Pathology and Medicine*.

[28] Myint M, Yuan ZN, Schenck K (2000). Reduced numbers of Langerhans cells and increased HLA-DR expression in keratinocytes in the oral gingival epithelium of HIV-infected patients with periodontitis. *Journal of Clinical Periodontology*.

[29] Feller L, Wood NH, Raubenheimer E (2006). Complex oral manifestations of an HIV-seropositive patient. *Journal of the International Academy of Periodontology.*.

[30] Lamster IB, Grbic JT, Bucklan RS, Mitchell-Lewis D, Reynolds HS, Zambon JJ (1997). Epidemiology and diagnosis of HIV-associated periodontal diseases. *Oral Diseases*.

[31] de Souza Gonçalves L, Ferreira SMS, Silva A, Villoria GE, Costinha LH, Colombo APV (2005). Association of T CD4 lymphocyte levels and chronic periodontitis in HIV-infected Brazilian patients undergoing highly active anti-retroviral therapy: clinical results. *Journal of Periodontology*.

[32] Vastardis SA, Yukna RA, Fidel PL, Leigh JE, Mercante DE (2003). Periodontal disease in HIV-positive individuals: association of periodontal indices with stages of HIV disease. *Journal of Periodontology*.

[33] Gonçalves LDS, Ferreira SM, Silva A (2004). Association of T CD4 lymphocyte levels and subgingival microbiota of chronic periodontitis in HIV-infected Brazilians under HAART. *Oral Surgery, Oral Medicine, Oral Pathology, Oral Radiology, and Endodontics*.

[34] de Souza Gonçalves L, Soares Ferreira SM, Souza CO, Souto R, Colombo APV (2007). Clinical and microbiological profiles of human immunodeficiency virus (HIV)-Seropositive Brazilians undergoing highly active antiretroviral therapy and HIV-Seronegative Brazilians with chronic periodontitis. *Journal of Periodontology*.

[35] Porter SR, Scully C, Luker J (1993). Complications of dental surgery in persons with HIV disease. *Oral Surgery Oral Medicine and Oral Pathology*.

[36] Dodson TB (1997). HIV status and the risk of post-extraction complications. *Journal of Dental Research*.

[37] Offenbacher S (1996). Periodontal diseases: pathogenesis. *Annals of Periodontology*.

[38] Takeichi O, Haber J, Kawai T, Smith DJ, Moro I, Taubman MA (2000). Cytokine profiles of T-lymphocytes from gingival tissues with pathological pocketing. *Journal of Dental Research*.

[39] Taubman MA, Kawai T (2001). Involvement of T-lymphocytes in periodontal disease and in direct and indirect induction of bone resorption. *Critical Reviews in Oral Biology and Medicine*.

[40] Spellberg B, Edwards JE (2001). Type 1/type 2 immunity in infectious diseases. *Clinical Infectious Diseases*.

[41] Monaco JL, Lawrence WT (2003). Acute wound healing: an overview. *Clinics in Plastic Surgery*.

[42] Park JE, Barbul A (2004). Understanding the role of immune regulation in wound healing. *American Journal of Surgery*.

[43] Luck JV, Logan LR, Benson DR, Glasser DB (1996). Human immunodeficiency virus infection: complications and outcome of orthopaedic surgery. *Journal of American Academy of Orthopedic Surgery*.

[44] Tappuni AR, Fleming GJP (2001). The effect of antiretroviral therapy on the prevalence of oral manifestations in HIV-infected patients: a UK study. *Oral Surgery, Oral Medicine, Oral Pathology, Oral Radiology, and Endodontics*.

[45] Shangase L, Feller L, Blignaut E (2004). Necrotising ulcerative gingivitis/periodontitis as indicators of HIV-infection. *South African Dental Journal*.

[46] Mortality and causes of death in South Africa. http://www.statssa.gov.za/PublicationsHTML/P030932005/html/P030932005.html.

